# Bidirectional Tendon Strip: A Rectus Femoris Graft Harvesting Technique for Anterior Cruciate Ligament Reconstruction

**DOI:** 10.1016/j.eats.2025.103916

**Published:** 2025-10-10

**Authors:** Jia Ying Lee, Jun Wei Soong, Deborah Huang, Paul Chang, Ting Cong, Matthieu Ollivier, Zi Yang Chia

**Affiliations:** a Department of Orthopaedic Surgery, Singapore General Hospital, Singapore, Singapore; b Department of Orthopaedic Surgery, University of Pittsburgh, Pittsburgh, Pennsylvania, U.S.A.; c Department of Orthopaedic Surgery, Aix-Marseille University, Marseille, France; d Duke-National University of Singapore Medical School, Singapore, Singapore; e Yong Loo Lin School of Medicine, National University of Singapore, Singapore, Singapore

## Abstract

In recent years, alternative graft options for anterior cruciate ligament (ACL) reconstruction have gained attention, with the quadriceps tendon and rectus femoris (RF) favored for their biomechanical robustness and biological integration potential. However, traditional retrograde techniques harvesting from the patellar insertion pose technical challenges. These include poor visualization, tight overlying soft tissues and condensation of the quadriceps tendons at the suprapatellar region, and increased risk of capsular breach and donor-site morbidity. We describe the bidirectional tendon strip technique, in which retrograde harvesting is initiated 8 cm proximal to the superior pole of the patella with an open stripper. This point allows for consistent identification of tendon width and depth with minimal dissection. We then proceed to harvest distally with a cylindrical tendon corer. This approach enables controlled graft harvesting with predictable thickness and length for a broad range of reconstructions, including ACL reconstruction with various lateral stabilization techniques and PCL reconstruction. The bidirectional tendon strip technique represents a refined, reproducible approach for RF tendon harvest in ACL reconstruction. It integrates the anatomic consistency and strength of the RF with a minimally invasive strategy that minimizes morbidity and optimizes graft characteristics.

Anterior cruciate ligament (ACL) reconstruction continues to evolve, with graft selection central to optimizing biomechanical performance while minimizing donor-site morbidity. Although bone–patellar tendon–bone and hamstring tendon grafts remain standard options with established outcomes, quadriceps tendon (QT) autografts have gained traction, particularly in young and athletic patients.

The QT is a tri-laminar structure formed by 6 components: lateral aponeurosis of the vastus intermedius, deep and superficial medial aponeuroses of the vastus intermedius, vastus lateralis, tensor vastus intermedius, and superficial rectus femoris (RF).^1^ Its multilayered architecture, large cross-sectional area, and lower donor-site morbidity confer strong biomechanical properties, with outcomes comparable to bone–patellar tendon–bone and hamstring tendon grafts.^2^

QT autografts may be harvested full or partial thickness, with or without a bone block. Partial-thickness grafts, favored for reduced donor-site morbidity and faster recovery,^2^^,^^4^ provide clinical outcomes similar to full-thickness grafts, but biomechanical studies show lower load-to-failure strength.^3^ To address this limitation, Strauss et al.^5^ described doubling the RF tendon, reporting superior pull-to-failure strength and cyclic stiffness compared with partial-thickness QT grafts.

The RF tendon offers several advantages^6^: It yields a long, robust graft suitable even for multiligament reconstruction, and superficial harvest spares the vastus medialis, deep vastus intermedius, and capsule. Biomechanically, it shows high tensile strength and stiffness comparable to or exceeding conventional grafts.^5^^,^^6^

Traditionally, retrograde RF harvest begins just above the superior pole of the patella (SPP), with the tendon dissected and removed proximally using a closed stripper.^7^ More recently, Panas et al.^8^ proposed antegrade harvesting via a horizontal incision approximately 8.5 cm above the SPP, allowing a more predictable graft length, avoidance of the SPP vascular plexus, and reduced risk of arthrotomy.

Building on these techniques, we describe a minimally invasive bidirectional tendon strip (BTS) technique, which begins 8 cm proximal to the SPP and allows both proximal and distal harvesting through a single incision. By combining the advantages of proximal initiation with enhanced control over graft length and depth (Table 1), the BTS technique aims to improve consistency, reduce donor-site morbidity, and overcome the limitations of current QT harvest methods.

**Table 1:** Comparison of Distal-to-Proximal and Proximal-to-Distal Quadriceps Tendon Harvest Techniques

Feature	Distal-to-Proximal Harvest	Proximal-to-Distal Harvest
Incision location	Just above patella	Approximately 8 cm above SPP
Visualization	Limited owing to tight distal tissues	Improved owing to soft-tissue laxity proximally
Risk of arthrotomy	Higher because of proximity to capsule	Lower if retinacular confluence is avoided
Donor-site morbidity	May increase anterior knee pain and hematoma	Reduced because of less trauma to muscle, capsule, and vasculature
Length control	May be imprecise with inaccurate starting point	Predictable based on initial entry
Technical difficulty	Challenging with tight distal space	More accessible; facilitates MIS approach

MIS, minimally invasive surgery; SPP, superior pole of patella.

## Surgical Technique

### Patient Positioning and Preparation

**Fig 1:**
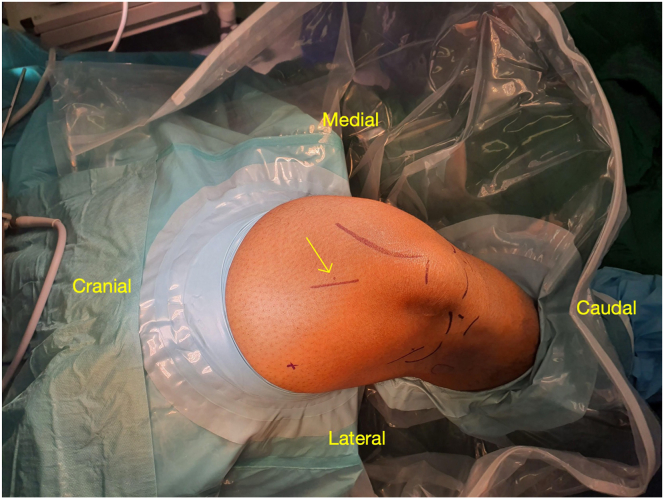
The patient is positioned supine with a right-side support and the right knee flexed to 90°; the foot is on a sandbag. The arrow points toward the marked 2-cm longitudinal incision centered 8 cm proximal to the superior pole of the patella.

The patient is positioned supine on the operating table (Video 1). A lateral post and foot holder are applied to stabilize the leg in 45° of knee flexion. A thigh tourniquet is placed as proximal as possible to ensure a bloodless field. The surgical leg is prepared and draped in the usual sterile fashion (Fig 1).

### Skin Incision and Soft-Tissue Dissection

A 2-cm vertical or transverse midline incision is made approximately 8 cm proximal to the SPP. Its medial-lateral position also references the vastus medialis muscle bulk. This 8-cm mark corresponds to a point of the RF tendon at which the soft-tissue envelope is more mobile and less densely packed than at the distal insertion. This is a mobile window, and the tendon can be brought into the view of this window by flexing and extending the knee. This approach facilitates a minimally invasive incision. This incision can be made before or after the scope portals are created. Fluid distension of the suprapatellar pouch can predispose to inadvertent arthrotomies in conventional QT harvesting. However, in this bidirectional technique, clear tissue planes at this proximal starting point—and a resultant well-preserved vastus intermedius—provide ample support against capsular breach.

The subcutaneous tissue is dissected down to the rectus tendon. In patients with a significant adipose layer, subcutaneous adipose tissue may require excision. Blunt dissection and Metzenbaum scissors are used to develop a plane around the tendon. Keeping the knee in 20° to 45° of extension is useful for this dissection. Large and small Langenbeck retractors can aid in visualization.

### Tendon Identification and Isolation

**Fig 2:**
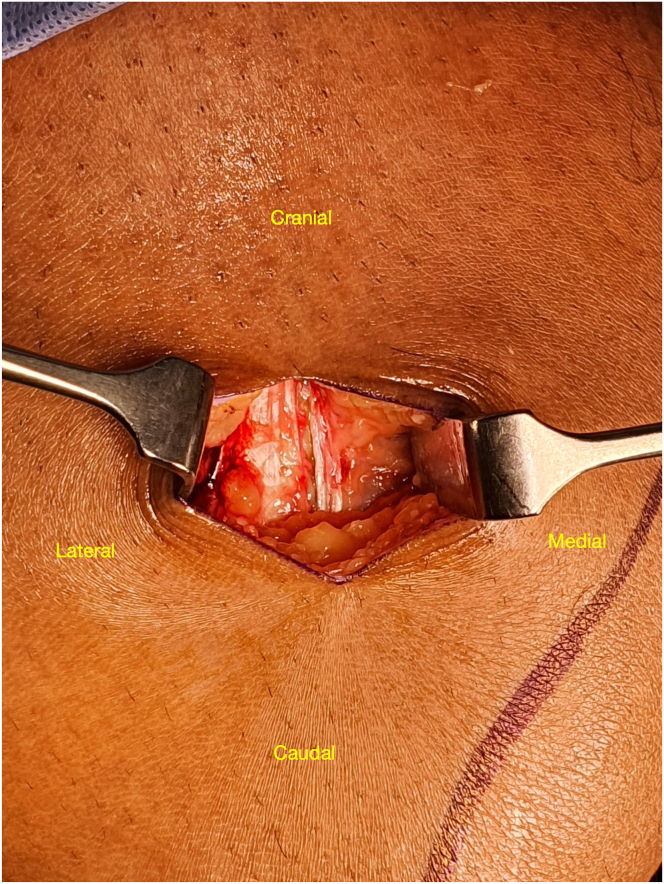
Right knee, supine position with knee flexed. After skin incision and fat excision at the thigh 8 cm from the superior patella pole, the rectus femoris tendon is seen in the right knee with a plane developed around it.

**Fig 3:**
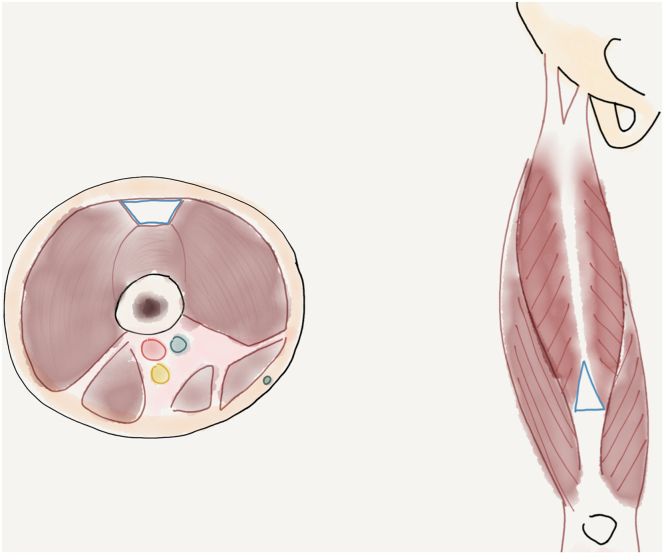
Fascial bands (blue triangle) in the segment of the rectus femoris harvest shown can compromise passage of the graft stripper. Blue trapeziod shows the fascial bands in cross section, red, blue and yellow circles demonstrate the popliteal artery, vein and sciatic nerve in the neurovascular bundle at the posterior thigh.

The RF tendon is identified by its glistening white appearance and location superficial to the vastus intermedius (Fig 2). At the 8-cm mark of the RF tendon, the vastus medialis is largely muscular. Part of it lies over the RF tendon, and this can be gently swept away with a Cobb elevator or blade. This allows better appreciation of the medial- and lateral-most extent of the RF tendon. Hence, the starting point of the parallel incision is consistently in the correct place in the medial-lateral axis. From this view, the trajectory of the tendon is easily assessed as well. In addition, the easy removal of bands from the vastus medialis and other QTs at this proximal starting point (Fig 3) reduces the risk of premature transection or stripper damage during passage of the stripper.

With a No. 15 blade, parallel incisions are made according to the desired graft thickness, with a precise assessment of the maximum RF width available. The blade is advanced to a depth limited by the fat separating the RF from the vastus intermedius. The tendon is gently elevated from the deeper fat plane overlying the rectus intermedius and mobilized using a right-angle clamp. The surgeon should ensure that the parallel incisions medial and lateral to the RF tendon are clear of bands using Metzenbaum scissors (especially proximally) to ease subsequent passage of the stripper. Care is taken to preserve the surrounding tissue and avoid postoperative hematoma formation.

### Bidirectional Harvesting

##### Retrograde (Proximal) Harvest

**Fig 4:**
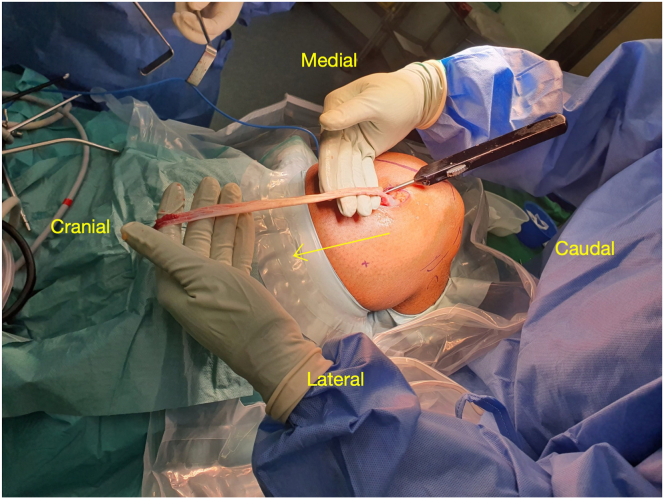
Retrograde rectus femoris harvest using Semitendinosus Tendon Stripper. The arrow shows the direction of advancement of the tendon stripper. The surgeon is holding the proximal end of the harvested rectus femoris tendon.

An open tendon stripper (Semitendinosus Tendon Stripper; Arthrex, Naples, FL) is introduced over the tendon. Using the tension from the intact rectus insertion and retraction from right-angle retractors or tape, the stripper is gently advanced proximally toward the anterior superior iliac spine (Fig 4). We have not encountered any difficulty with passing the stripper under an inflated tourniquet. With experience, harvesting can be performed tourniquet free.

##### Antegrade (Distal) Harvest

**Fig 5:**
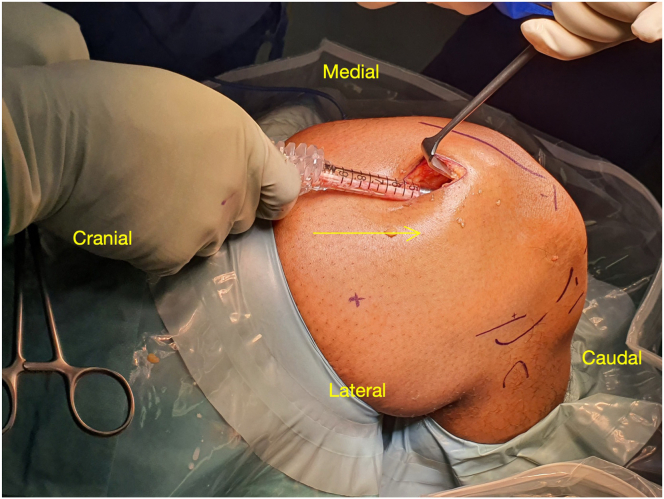
Antegrade rectus femoris harvest with QuadPro Tendon Harvester Graft Harvesting System. The arrow shows the direction of advancement of the QuadPro system.

A cylindrical tendon corer (e.g., QuadPro Tendon Harvester Graft Harvesting System; Arthrex) is inserted through the same incision and directed distally. The corer is advanced under direct palpation and trajectory control, harvesting an additional 7 to 9 cm of graft length depending on patient anatomy (Fig 5). Skewing slightly medially also helps to maintain a partial-thickness harvest. Once the desired length is achieved, the graft is truncated. This segment provides a robust, broad portion of the graft. No sutures need to be passed into the graft during the entire harvesting process, which also increases the efficiency of this technique.

### Graft Preparation

**Fig 6:**
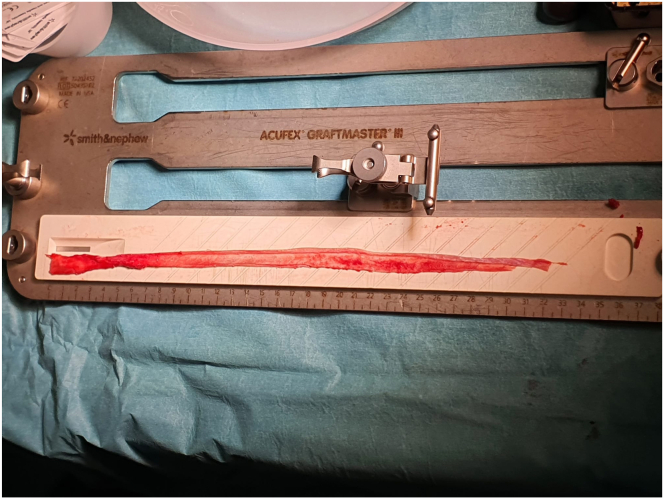
The length of the rectus femoris graft, shown on a graft board, is 32 cm.

**Fig 7:**
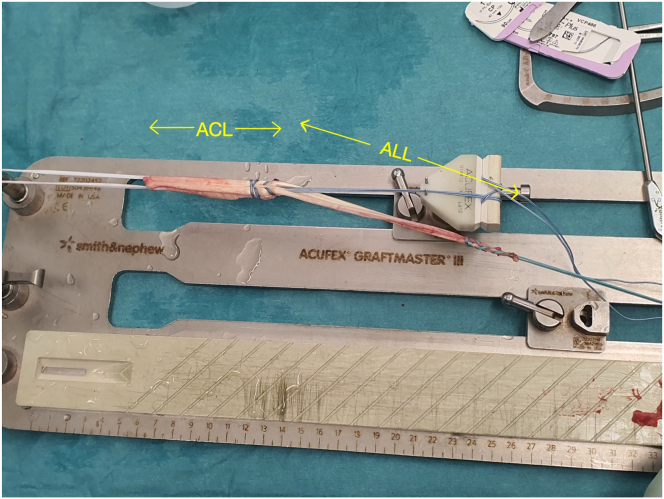
Rectus femoris graft prepared for combined anterior cruciate ligament (ACL) and anterior longitudinal ligament (ALL) reconstruction.

The harvested graft is cleaned of residual muscle tissue. A typical graft measures 8 to 11 mm in diameter and 28 to 35 cm in length (Fig 6). The distal end tends to be thicker and stronger, whereas the proximal end offers sufficient length for versatile graft configurations. Depending on surgical preference, the graft may be further augmented with suture tape or prepared as a dual-bundle construct for ACL/anterolateral ligament or ACL/lateral extra-articular tenodesis or double-bundle ACL reconstructions (Fig 7).

### Closure

The tendon harvest site is irrigated, and hemostasis is achieved. Because this approach allows for consistent identification of depth, partial-thickness graft harvests are to be expected, hence sparing the need for closure of the quadriceps or capsular layer. The subcutaneous and skin layers are closed in layers. A sterile dressing is applied. Pearls and pitfalls of our method are summarized in Table 2.

**Table 2:** Pearls and Pitfalls of BTS Harvesting

	Pearls	Pitfalls
Approach	The knee should be flexed or extended to visualize the tendon within the view of the mobile skin window. The vastus medialis muscle bulk should be referenced to the site incision over the RF tendon. Insulated tip diathermy should be used to protect against dermal injury.	If arthroscopy is performed prior to harvest, fluid from the knee joint should be milked to minimize capsular distension.
Dissection	Blunt dissection should performed to free adipose tissue and the overlying vastus medialis to see the full width of the RF tendon.	Gentle sharp dissection is performed, stopping at the depth of fat delineating the plane between the RF and VI. Breaching this plane can lead to inadvertent arthrotomy. The surgeon should not stop at the delaminations within the RF, which can result in harvesting a graft that is thinner than intended.
Harvest	With traction on the tendon via tape or a right-angle clamp, the bands are released, especially medially. This will ease passage of the stripper proximally and the corer distally. The surgeon should switch to an adult-sized retractor on the “roof” of the dissection for improved visualization in MIS incisions. To maintain intact deeper layers, there is no need to fill the corer completely.	The surgeon should direct the corer distally, erring medially, and in the axis of the femur to keep the VI intact and avoid capsular breach. Knee flexion keeps the tendon taut, facilitating tendon transection. Gentle placement of an open stripper is performed over the RF to avoid damaging the tendon, as this is the usual apex zone for hinging suspensory fixation.

BTS, bidirectional tendon strip; MIS, minimally invasive surgery; RF, rectus femoris; VI, vastus intermedius.

## Discussion

The BTS technique offers several key advantages over conventional QT or RF harvesting methods. By combining proximal initiation with controlled bidirectional dissection, this technique addresses many of the anatomic and technical limitations associated with traditional harvest approaches. By initiating the harvest approximately 8 cm proximal to the SPP, the surgeon operates in a region with decreased fascial resistance and greater tendon mobility. The RF is at its narrowest width 5 cm proximal to the SPP.^6^ This proximal starting point enables consistent identification of tendon depth and maximal width, allowing for more precise and controlled graft preparation. It has been theorized that the synovial side of the QT predisposes it to a higher rate of cyclops formation. The partial-thickness nature of the RF removes this risk.

The reduced tension in the proximal soft tissue further enhances visualization and maneuverability, supporting minimally invasive techniques. This decreased wound tension over the thigh compared with the SPP (which overlies the knee joint and has higher tension in flexion) also reduces the risk of wound-related complications such as hypertrophic scarring or keloid formation and yields a better esthetic appearance. This complements the smaller incision that improved visualization facilitates.

The average length of the QT has been reported to be 49 ± 7 mm in women and 50 ± 9 mm in men,^1^ whereas the RF tendon graft typically yields a length of 28 to 35 cm. The resulting graft is suitable for a wide range of reconstructions, including ACL and anterolateral ligament reconstruction (over the top), ACL reconstruction and lateral extra-articular tenodesis, and double-bundle reconstruction; over-the-top techniques^9^; and revision surgery—offering surgeons a single harvest site for multipurpose graft use, as well as fixation preferences. Surgeons have an option of avoiding the patellar insertion zone to reduce the risk of violating the capsule and minimizing postoperative extravasation, anterior knee pain, and hematoma. Removing the need for quadriceps closure also improves the efficiency of the procedure. In their description of a purely antegrade harvest technique, Panas et al.^8^ mentioned that they routinely close the defect. It is likely that this unidirectional harvest requires a thicker graft to compensate for short length, hence the resultant larger defect, which causes surgeons to favor closure. The bidirectional harvesting circumvents this drawback by harvesting a good length of tendon proximally as well, reducing the thickness requirement distally. Harvesting in both directions provides flexibility in tailoring the graft length and thickness, ensuring optimal fit for single-ligament or multiligament reconstructions. This is also useful for petite female patients who are predisposed to having short thin grafts. In the rare situation in which the distal tendon is sufficiently thick and the graft requirements have been met, proximal harvest can be omitted. Some techniques use a bone block for its secondary advantage in extending the length of the graft. The consistent length of this graft obviates this requirement. The advantages and disadvantages of the BTS technique are summarized in Table 3.

**Table 3:** Advantages and Disadvantages of BTS Technique

	Explanation
**Advantages**	
Technically efficient	This technique is highly reproducible across patient morphologic types. Given its low technical requirements, this technique is accessible to surgeons with varying experience in ACL surgery. There is also no need for repair of the donor site.
Fast patient rehabilitation	By consistently delivering a partial quadriceps tendon harvest, sparing the vastus intermedius and medialis and capsule, donor-site morbidity is minimal.
Versatile graft	Because the RF graft is long and substantial, it can be prepared in multiple configurations for various ACL and/or PCL reconstruction constructs, with graft to spare for lateral stabilization procedures.
**Disadvantages**	
Limited access to SPP	Because the BTS technique avoids exposure to the superior patellar pole, subsequent surgical access for direct capsule repair should there be a breach in the capsule may be restricted. Although not encountered in our experience, suture passer instruments may be used for deep distal repair.
Lack of bone-to-bone healing	For surgeons who prioritize bone-to-bone healing, the bone block is not accessible via this approach.
Lack of long-term outcome data	Prospective clinical and biomechanical studies are needed to evaluate durability, graft incorporation, and patient outcomes, especially in relation to extensor recovery.

ACL, anterior cruciate ligament; BTS, bidirectional tendon strip; PCL, posterior cruciate ligament; RF, rectus femoris; SPP, superior pole of patella.

Overall, the RF BTS technique maintains the advantages of a quadriceps-based graft and preserves the hamstrings, which are agonists of the ACL; this assists in preventing anterior translation of the tibia. By leveraging a midpoint incision and combining retrograde and antegrade tools, this technique addresses the limitations of traditional distal-to-proximal quadriceps harvest approaches while maximizing graft quality and consistency. This simplified approach promises to accelerate the learning curve of surgeons, and it reduces patients’ morbidity. The technique’s versatility makes it advantageous for complex reconstructions requiring extended graft length, permitting surgical flexibility, graft customization, and improved muscle recovery. Although biomechanical testing and prospective outcome studies are necessary to validate the long-term clinical utility of this technique, the BTS technique presents a promising and biologically favorable option for ACL reconstruction.

## Disclosures

All authors (L.J.Y., S.J.W., D.H., P.C., T.C., M.O., C.Z.Y.) declare that they have no known competing financial interests or personal relationships that could have appeared to influence the work reported in this paper.

## Supplementary Data

Video 1 Bidirectional Tendon Strip (BTS) technique for easy rectus femoris tendon harvesting.
